# Inno4Vac Workshop Report Part 2: RSV‐Controlled Human Infection Model (CHIM) Strain Selection and Immune Assays for RSV CHIM Studies, November 2021, MHRA, UK

**DOI:** 10.1111/irv.70013

**Published:** 2024-10-23

**Authors:** Joanna Waldock, Rebecca J. Cox, Othmar G. Engelhardt, Stephanie Ascough, Albert Osterhaus, Guus F. Rimmelzwaan, Martin Ludlow, John S. Tregoning, Jacqueline U. McDonald, Ursula J. Buchholz, Rienk E. Jeeninga, Charles. Sande, Christopher Chiu

**Affiliations:** ^1^ Influenza Resource Centre, Vaccines, Science Research & Innovation Medicines and Healthcare Products Regulatory Agency Potters Bar UK; ^2^ Influenza Centre, Department of Clinical Sciences University of Bergen Bergen Norway; ^3^ Department of Infectious Disease Imperial College London London UK; ^4^ Research Centre for Emerging Infections and Zoonoses University of Veterinary Medicine Hannover Hannover Germany; ^5^ RNA Viruses Section, Laboratory of Infectious Diseases National Institute of Allergy and Infectious Diseases (NIAID) Bethesda Maryland USA; ^6^ Viroclinics Biosciences B.V. Rotterdam The Netherlands; ^7^ KEMRI‐Wellcome Trust Research Programme Kilifi Kenya; ^8^ Centre for Tropical Medicine & Global Health University of Oxford Oxford UK

**Keywords:** controlled human infection model (CHIM), correlates of protection (CoP), immune assays, respiratory syncytial virus (RSV), respiratory viruses

## Abstract

Controlled human infection models (CHIMs) are a critical tool for the understanding of infectious disease progression, characterising immune responses to infection and rapid assessment of vaccines or drug treatments. There is increasing interest in using CHIMs for vaccine development and an obvious need for widely available and fit‐for‐purpose challenge agents. Inno4Vac is a large European consortium working towards accelerating and de‐risking the development of new vaccines, including development of CHIMs for influenza, respiratory syncytial virus and *Clostridium difficile* . This report (in two parts) summarises a workshop held at the MHRA in 2021, focused on how to select CHIM candidate strains of influenza and respiratory syncytial virus (RSV) based on desirable virus characteristics and which immune assays would provide relevant information for assessing pre‐existing and post‐infection immune responses and defining correlates of protection. This manuscript (part 2) summarises presentations and discussions centred around RSV CHIMs and immune assays (an additional manuscript summarises influenza CHIM and immune assays: Inno4Vac workshop report Part 1: Controlled human influenza virus infection model (CHIVIM) strain selection and immune assays for CHIVIM studies, November 2021, MHRA, UK).

## 1 Introduction

The development of controlled human infection models (CHIMs) forms part of a large programme of work in the Inno4Vac consortium focused on accelerating and de‐risking the development of new vaccines. There are several advantages to using a challenge model over natural infection during clinical trials, such as increased reproducibility and robustness of results, known time of infection, known virus isolate, controlled infection dose and pre‐screening of individuals with known risk factors. A challenge model provides a well‐controlled study to obtain Phase 1 or 2b data for vaccines and antivirals. Following on from a previous report summarising an expert workshop held at the MHRA in November 2021 focusing on influenza CHIVIM virus strain characteristics (Inno4Vac workshop report Part 1: Controlled human influenza virus infection model (CHIVIM) strain selection), this report summarises the presentations and discussions around respiratory syncytial virus (RSV) CHIM strain characteristics and immune assays for CHIM studies (assessing both pre‐existing immunity and post‐infection immunity during CHIM studies using RSV).

## 2 RSV Strain Characteristics for CHIM Development

### 2.1 Recently Developed RSV CHIM Strains

Some of the considerations for the selection of RSV strains are less complex than those for influenza, although there are still many factors that should be taken into account when selecting a strain—this was the focus of the second session of the workshop. RSV has two antigenic and genetic subgroups, A and B. The reference genomes of the two subgroups share 81% nucleotide identity, and there is a high degree of genome‐wide similarity or identity in nucleotide or amino acid sequence between subgroups A and B, with greater divergence in the attachment protein (G) (53% amino acid identity between the reference genomes) and greater similarity (89% amino acid identity) in the fusion protein (F). The subgroups exhibit a three‐ to fourfold reciprocal difference in neutralisation by subgroup‐specific antisera [1], and there is substantial immune cross protection, mainly based on the F protein. F is considered the primary antigenic target of the virus [2], with G as a secondary target. Both the A and B subgroups have acquired a nucleotide duplication in the C‐terminal region of the G gene, first identified in the subgroup B strains in 1999 and in the subgroup A strains in 2010 [3]. Within each subgroup, there are multiple genotypes (minimum 13 subgroup A and 20 subgroup B genotypes) [4]. At the time of the workshop, no effective antivirals for RSV were available, but multiple vaccines for the elderly were under development, as well as passive approaches (long half‐life monoclonal antibodies or protein subunit vaccines for maternal immunisation in the last weeks of pregnancy). Two protein subunit vaccines for the elderly and two passive‐protection products for neonates and infants have been licensed in the meantime (Medicines and Healthcare products Regulatory Agency authorises GSK's Arexvy, the first RSV vaccine for older adults |GSK, Beyfortus approved in the US for the prevention of RSV lower respiratory tract disease in infants (astrazeneca.com), last accessed 15/11/2023).

The session began with a summary of a previously developed RSV CHIM, A/Maryland/001/11. In 2013, the NIH decided to develop an RSV challenge strain that could be used in healthy volunteers with pre‐existing immunity, which would be made available for collaborators to study new antivirals and vaccines. The NIH aimed to characterise safety, infectivity, and immunogenicity of this RSV challenge strain based on an at the time recent RSV isolate. In 2011, during a clinical study, an RSV (genotype GA) strain was isolated from a participant with naturally acquired infection [5]. The virus was isolated and passaged once prior to sequencing (no evidence of cell‐culture adaptive mutations was observed). Challenge material of this RSV A/Maryland/001/11 was generated using a reverse genetics (RG) system, generating a replication competent RSV that contains a codon‐optimised G ORF (to stabilise the cDNA for replication in bacteria). Compared to the previous RSV/A challenge strain (RSV‐A Memphis‐37b) [6], this recombinant virus was most divergent in the G protein (even though it did not contain the G duplication), likely changes driven by immune pressure, and in the M2‐2 protein. The RG approach generates very clean material: A single passage on qualified Vero cells yielded clinical trial material that is free of recombinant DNA and adventitious agents with a brief, well‐defined, passage history. The virus replicated in primary human epithelial airway cells efficiently and was comparable to RSV A2 in pre‐clinical safety studies in African green monkeys. A first‐in‐human study was carried out on an inpatient basis to determine safety and infectivity of the challenge agent, to quantify virus shedding over time and to investigate immunogenicity through measurement of serum IgG to RSV F and plaque reduction neutralisation tests (PRNT_60_). Inclusion criteria for the CHIM were healthy males and non‐pregnant females 18–50 years of age. RSV serum neutralising antibodies were additionally measured, with the lower 50% of the range included in the study. The details of the study can be accessed online (Inpatient Challenge Study of rRSV A/Maryland/001/11, a Human Respiratory Syncytial Virus Challenge Strain, Administered to Healthy Adult Volunteers ‐ Full Text View ‐ ClinicalTrials.gov last accessed 15/11/2023). Participants were given a challenge dose of 10^5^ plaque‐forming units (PFU). Seventy‐five per cent of participants experienced symptomatic infection, with no lower respiratory tract infections or serious adverse events (SAEs). Forty‐five per cent of participants had challenge virus detectable in nasal wash specimens by immunoplaque assay, with peak virus shedding detected around Day 4 post‐infection. Some participants that were negative by culture were positive by RT‐PCR; however, symptomology was much reduced in participants that were positive by RT‐PCR only compared to those positive by culture and RT‐PCR. In several participants, RSV was detectable in nasal wash specimens for a prolonged period. Further work on this trial was severely impaired by the COVID‐19 pandemic—a further two cohorts were due to be enrolled in the study in 2020, but this was put on hold. Additional analysis of peripheral blood mononuclear cells (PBMCs) and antibodies is ongoing. Future studies will include higher and lower challenge doses to determine optimum dose for safe symptomatic disease. The challenge virus preparation is available under US FDA IND for evaluation of antivirals or vaccines in adult volunteers.

The next talk outlined another RSV pipeline for CHIM development to support early phase trials by developing an RSV CHIM strain in Europe. RSV‐A and RSV‐B isolates were obtained from juvenile patients that could be used for human challenge. Additionally, reverse genetics (RG)–derived molecular clones of these isolates were also made—this combination of WT and accompanying molecular clones is unique [7]. An RSV‐B strain (BA9 genotype) was isolated and passaged in HEp‐2 cells to assess genetic stability [7]. This strain was used to generate a GMP virus stock in MRC‐5 cells. At the time of the workshop, post‐production GMP testing and in vivo toxicity and safety data was ongoing prior to GMP virus release and regulatory approval for a dose escalation trial.

### 2.2 Global Considerations for RSV CHIM Studies

The next talk focused on the global considerations for selecting an RSV challenge agent. There is considerable global diversity in RSV strains, and rapid worldwide spread of new strains is observed such as the emergence of RSV‐A strains containing a sequence duplication in the G gene, which were first identified in 2010 and found worldwide by 2014 [8]. However, differences in disease risk and disease profile are apparent in distinct geographical regions. This is influenced by the numbers of people within a population at high risk of severe RSV infection such as HIV‐positive individuals and older adults or the role of household air pollution in increasing the risk of severe respiratory disease in vulnerable populations.

HIV‐positive populations have a particularly high risk of severe RSV disease. The incidence of hospitalisation with RSV disease has been shown to be around 20 times higher in HIV‐positive people, irrespective of age [9]. HIV‐positive individuals are an important at‐risk group; however, there is little understanding of the underlying mechanism leading to increased pathology in this group. Genetic analysis of RSV strains from HIV‐positive children have shown within‐host evolution appears to be affected, with G protein deletions detected that have possible fitness implications [10]. Although these strains are unlikely to be transmissible to immunocompetent individuals, these highly defective viruses are apparently still able to replicate in HIV‐positive individuals and cause severe disease. A question for RSV CHIMs is whether current RSV CHIM models are translatable to HIV—currently data suggest this is likely not the case. A second question is whether studies in HIV+ individuals with controlled infection are feasible. This would likely depend on the relative risk of severe adverse events (SAEs) and availability of a rescue therapy.

RSV CHIM studies conducted in high‐income countries (HICs) likely do not account for chronic respiratory risk factors that other parts of the world experience. A specific example of this is the effect of household air pollution on the risk of developing severe RSV disease. Around 50% of the world's population are regularly exposed to household air pollution through incomplete combustion of fuels. This high level of exposure to smoke pollution has been known to modify the pathology of RSV [11]. It is not possible to replicate this risk factor in RSV CHIMS in HICs. Household air pollution affects the lung microbiome of healthy adults. Alveolar macrophages phagocytose particulate matter from household smoke, and chronic exposure leads to continuous low‐grade inflammation in the lungs [12]. This may account for the increased morbidity and mortality from RSV disease. In mice, exposure to black carbon increases neutrophilia, decreases lymphocytes and alters cytokine expression during RSV infection [13]. People with chronic exposure to smoke pollution are likely to experience RSV infection in a different way due to this low‐grade inflammation in the lungs. It is not possible to replicate this risk factor in RSV CHIMs in HICs. CHIM in populations representing 50% of the world's population who are exposed to household air pollution would help to identify the mechanisms of severe RSV disease.

There is a long history of CHIMs in Europe and the United States, but this is a fairly novel concept outside HICs. In recent years a malaria CHIM has been established in Kenya [14], in Malawi a pneumococcus CHIM has been established [15], and Shigella [16] and Schistosomiasis (Human Infection Studies for Schistosoma mansoni (CHI‐S) in Uganda | LSHTM last accessed 15/11/2023) models are being set up in Kenya and Uganda, respectively. There is increasing acceptance and trust within the community allowing these studies to be set up. These studies have used clinical isolates of pathogens that have previously been characterised in HIC and are relevant to local populations in terms of disease burden. Areas of concern where acceptance may be lower are use of challenge strains derived by reverse genetics (e.g., in Kenya there is a ban on genetically modified organisms [GMOs]), the immaturity of the regulatory environment (where it is advisable to co‐ordinate with regulators at an early stage of CHIM development and incorporate their comments and requirements from the outset and build confidence and address concerns) and the limited scope for rescue therapies and challenges with high level supportive care.

### 2.3 RSV Strain Characteristics That Are Desirable for a CHIM Agent

The final talk of the session summarised RSV challenge agent strain criteria desirable for a CHIM. The intended use of the CHIM is important and may affect the criteria used for selection of the strain. The Inno4Vac consortium aims to develop an RSV CHIM for use in accelerating vaccine development. More contemporary RSV CHIMs are required as currently no B strain is available and the currently available RSV‐A strain CHIM agents do not contain the G protein duplication found in recent circulating strains [17]. The choice of an RSV‐A or RSV‐B strain has been widely debated, with advantages and disadvantages on both sides, for example, RSV‐A strains grow to higher titres making manufacture and use in immune assays more straightforward. However, the lack of an RSV‐B strain CHIM represents a significant gap in the RSV CHIM landscape.

Characteristics that are important for strain selection are:

- Contemporary strain—currently available RSV CHIM strains are from 2007 (RSV A Memphis‐37) and 2011 (RSV A/Maryland/001/11)
- Should be representative of clinical strains that are circulating
- Documentation of disease symptoms/severity of disease
- Relevant consent in place for development of strain into a CHIM
- Documentation of method of isolation and production (e.g., avoiding Vero passaged viruses due to the well documented issue concerning truncation of the G protein)
- Propagation history should be well documented with low passage numbers
- Growth characteristics defined in in vitro cell systems and human airway systems, with a titre of ~10^5^ PFU per mL required as a minimum—this could be a limiting factor for RSV‐B strains as they often grow to reduced titres in cell culture compared to RSV‐A strains
- Wide availability/accessibility—avoid limitations on the use of the strain after manufacture

Other issues for consideration include:

- Use of a clinical isolate or molecular clone (generated by RG)—these are genetically identical; however, there may be some issues with regulatory approval for use for molecular clones in certain countries. A clinical isolate with matched molecular clone is a useful tool: The molecular clone can be used as genetic background for mutations; additionally, neutralisation assays and T‐cell assays are facilitated by availability of a matched molecular clone (potentially including reporter genes such as GFP). Molecular clones also offer other advantages: less adventitious agent testing; ability to select the exact sequences you want; no adaptations to cell culture; no subpopulations.
- Retention of infectivity of a selected strain over time upon freezing—does a stabiliser need to be used? For example, sucrose, glycerol and fetal bovine serum (FBS). The choice of stabiliser is limited by requirements specific for clinical study material and may have consequences with respect to virus titre and toxicity for specific assays.

A review of existing RG systems for RSV shows that many were derived from strains that are relatively old or have an incomplete passage history and many of the plasmids containing a cDNA copy of the full‐length RSV antigenome are extremely unstable. Recently, a reverse genetics system has been established for contemporary isolates of RSV [7] using a bacterial artificial chromosome (BAC) as a vector backbone and has generated recombinant RSV‐A and RSV‐B strains based on clinical isolates obtained from RSV infected children. These recombinant RSVs contain the duplicated sequence in the G gene that is present in contemporary RSV‐A and RSV‐B strains, and the genetic sequence was stable after one passage on HEp‐2 cells.

## 3 Immune Assays for RSV CHIM Studies

### 3.1 RSV Antibody Assays

The next session focused on the standardisation of existing and development of new RSV antibody assays. A talk on the development of the first RSV international standard (IS) was given. Serum neutralising antibodies alone are known to confer a degree of protection from lower respiratory tract infection, and this measure is therefore the most commonly used correlate in RSV vaccine development. Although many disparate formats of virus neutralisation assay have been developed to measure these antibodies, recent development of a WHO international serum standard has enabled alignment of data from different laboratories and standardisation of results. The WHO Expert Committee on Biological Standardization established a serum pool (NIBSC product code 16/284) as the first international standard for antiserum to RSV in October 2017, with product 16/322 to be kept in reserve as a possible future replacement. They were assigned potencies of 1000 and 960 IU/ampoule, respectively. Both 16/284 and 16/322 have been shown to reduce inter‐lab variability of RSV‐A and RSV‐B neutralisation assays, including those with and without complement, making these assays comparable [18, 19]. The international standard has not been established to reduce variability of other serological assays. Regulators may require neutralising antibody titres to be expressed using the international standard, and vaccine manufacturers have been purchasing the standard if this becomes a requirement.

The next talk focused on development of RSV antibody assays. The RSV F protein is the major antigenic target for vaccine‐induced immunity as it is highly conserved across strains. This protein is now recognised to exist in multiple conformations, with the pre‐fusion F protein (pre‐F) understood to display epitopes against which the most potent neutralising antibodies are directed [20]. Virtually, all RSV vaccine candidates in recent development are therefore based on stabilised pre‐fusion F protein constructs. It is assumed that targeting vaccine‐induced immune responses towards epitopes that are uniquely displayed on the pre‐fusion form of F will lead to improved protective efficacy. Although neutralisation assays should largely correlate with pre‐F‐specific antibodies, this may differ between vaccine constructs and assays to measure pre‐F‐specific antibodies directly (viz., F protein competition ELISAs that can measure IgG, IgA or IgM) have been developed. Competitive ELISA using pre‐ and post‐F has been used to show robust boosting of serum IgG against the pre‐F protein after vaccination with a stabilised prefusion RSV F subunit protein candidate vaccine [20, 21]. As RSV‐specific nasal IgA has been shown to be a superior correlate of protection, in young healthy adults, compared to serum IgG [22], this competition assay was adapted for detecting IgA responses in nasal washes following infection with RSV Memphis‐37 strain [23]. An optimisation exercise revealed nasal washes to be a difficult matrix considering the immunological history of repeated exposure to RSV from a young age. No commercial negative controls for nasal wash exist, and quantities of true negative matrix (i.e., nasal washes from neonates) are typically low volume or difficult to acquire. Standardised positive controls for this matrix are also not readily available due both to the complexity of acquiring samples and the heterogeneity of RSV‐specific IgA seen in the general population. Optimisation identified repeated freeze–thaw cycles as particularly detrimental to protein stability for pre‐F‐specific ELISA. The optimised assay identified differential IgG and IgA responses to pre‐F between younger and older adults, where younger adults have a robust serum IgG and nasal IgA response after infection but older adults were boosted for serum IgG only whilst nasal IgA levels remained unchanged [23]. The F protein competitive ELISA is a very complex assay and is not likely to be used over neutralisation assays in the near future; however, the Inno4Vac project offers an opportunity to compare competition ELISAs with neutralisation assays side‐by‐side using samples from CHIM studies for measuring potential correlates of protection. Little standardisation of these assays has occurred between laboratories, with no central source of essential reagents such as recombinant proteins, and therefore, likely substantial inter‐laboratory variability exists. Because the quality of the antigen is a critical component of the assay, a reliable source and quality control steps to assess pre‐F and post‐F proteins will be necessary. Importantly, pre‐F‐specific antibody titres have not been definitively shown as a better predictor of efficacy than neutralising antibody.

### 3.2 Cell‐Mediated Immunity Assays

Whilst neutralising antibodies have been shown to be correlates of protection, there is growing interest in cell‐mediated immune effector mechanisms and their potential role in disease severity and duration. Mouse models show depletion of CD4 and/or CD8 cells reduces viral clearance, and adoptive transfer of Trm CD8+ T cells enhances vial clearance in primary/secondary infections [24, 25]. In humans, patients with CMI deficiencies have prolonged viral shedding and often high rates of severe disease and mortality [26]. Challenge studies have demonstrated that pre‐existing CD4+ T cells confer lower viral shedding and less severe illness during influenza infection [27], and the 2009 influenza pandemic revealed that pre‐existing T cells to conserved CD8 epitopes were correlated with less severe illness [28].

Several assays can be used for identifying pathogen‐specific T cells. In this workshop, an ELISPOT assay and multi‐parametric flow cytometry were discussed, specifically within the context of testing study subjects in an RSV‐controlled infection trial. IFN‐γ ELISPOT assays offer a robust, however somewhat crude, detection method for virus‐specific T cells. This type of assay may be useful in identifying non‐responder participants, reducing the number of samples to be further analysed using multi‐parametric flow cytometry. The peptide pools used for stimulation of PBMCs remain a concern, with commercially available peptides for RSV mostly covering the F protein and being from limited HLA types. Ideally, an overlapping peptide pool covering the whole viral proteome would be used; however, the cost of such a pool may be prohibitive. Multi‐parametric flow cytometry can be used to define the populations of T cells responding to infection within a CHIM, identifying CD4+ and CD8+ T‐cell phenotypes: naïve versus memory subsets; activation markers (e.g., CD38, HLA‐DR and CD137); CD4+ T follicular helper cells (CXCR5, PD‐1); exhaustion/senescence (CD57, KLRG1, PD‐1, TIM‐3); cytotoxic activity (GrxB, CD107a); polyfunctional T cells (Th1/Th2 cytokine profiles); and proliferation. Some important issues that require attention when measuring T‐cell responses centre around the fact that correlates of protection remain undefined. There is currently no idea of what numbers or what properties of T cells may afford protection. From this perspective, exploratory and in‐depth analysis from CHIM studies will provide valuable datasets for identification of CoPs.

### 3.3 Prioritising Immune Assays for Use in CHIM Studies

A final discussion session focused on prioritising immune assays within the Inno4Vac consortium for a planned RSV CHIM. The aim of this project is to develop an early‐phase clinical platform, including immunological parameters predicting field efficacy, which would allow (in future) the use of correlates of protection to predict vaccine candidates most likely to be efficacious that should go into field efficacy studies. It was recognised that, with limited resources, there will be a need to be pragmatic and focus only on standardising/harmonising those assays that can be commonly implemented for screening and analysis. There was consensus that some assays could be centralised for maximum efficiency so that work was not replicated across different sites. However, the small size of the planned studies encouraged the group of the possibility of running a wider range of assays. For today's vaccines, serological correlates of protection are top of the priority list. However, there is a need to consider the future and next‐generation vaccines. With the challenge model, there is a unique opportunity to assess assays that may not be used in Phase III clinical trials but can progress understanding of the disease and immune protection. Biobanking is therefore critical. A proposed pipeline for the Inno4Vac consortium was to conduct serum serologic analysis, investigate mucosal immunity and study T‐cell immunity initially screening using INF‐γ ELISPOT followed by further characterisation using multi‐parametric flow cytometry.

## 4 Conclusions and Closing Remarks

This workshop highlighted the existing gaps in the RSV CHIM landscape, with a lack of contemporary strains and no RSV‐B CHIM strain available to date. These discussions will inform decision‐making within the Inno4Vac consortium, which aims to address these gaps and develop a widely available RSV CHIM. The workshop also highlighted the need to use standardised immune assays in CHIM studies to better understand immune responses to infection and help define correlates of protection.

## Author Contributions

Conceptualisation: R.J.C., O.G.E. and C.C. Presentations/discussions: J.W., R.J.C., O.G.E., S.A., A.O., G.R., M.L., J.S.T., J.U.M., U.J.B., R.E.J., C.S. and C.C. Writing manuscript: J.W. Manuscript reviewing and editing: J.W., R.J.C., O.G.E., S.A., A.O., G.R., M.L., J.S.T., J.U.M., U.J.B., R.E.J., C.S. and C.C.

## Conflicts of Interest

J.W., R.J.C., O.G.E., S.A., G.R., M.L., J.S.T., J.U.M., U.J.B., C.S. and C.C. declare no conflicts of interest. R.E.J. was an employee of Viroclinics. A.O. is the CSO of CR2O.

### Peer Review

The peer review history for this article is available at https://www.webofscience.com/api/gateway/wos/peer‐review/10.1111/irv.70013.

## Data Availability

No new data was generated during this study.
